# Foetal Metformin Exposure, Childhood Adiposity and Future Cardiovascular Risk: Can We Connect the Dots?

**DOI:** 10.17925/EE.2025.21.2.4

**Published:** 2025-10-24

**Authors:** Simran Thakkar, Saptarshi Bhattacharya, Lakshmi Nagendra, Nitin Kapoor, Deep Dutta, Sanjay Kalra

**Affiliations:** 1. Department of Endocrinology, Indraprastha Apollo Hospitals, New Delhi, India; 2. Department of Endocrinology, JSS Medical College, JSS Academy of Higher Education and Research, Mysore, India; 3. Department of Endocrinology, Diabetes, and Metabolism, Christian Medical College and Hospital, Vellore, Tamil Nadu, India; 4. Non-communicable Disease Unit, Baker Heart and Diabetes Institute, Melbourne, Victoria, Australia; 5. Department of Endocrinology, Center for Endocrinology, Diabetes, Arthritis & Rheumatism, New Delhi, India; 6. Department of Endocrinology, Bharti Hospital, Karnal, Haryana, India

**Keywords:** Cardiovascular disease, genetic epigenesis, gestational diabetes, metabolism, metformin, paediatric obesity, prenatal exposure delayed effects, short-for-gestational age, type 2 diabetes

## Abstract

The prevalence of gestational diabetes (GD) and pre-existing diabetes during pregnancy has been increasing. Insulin is the accepted pharmacological treatment for hyperglycaemia in pregnancy. Metformin has emerged as a promising alternative or adjunct due to its ease of use, lower cost and reduced risk of hypoglycaemia. Beyond GD, metformin also shows potential utility in early GD and polycystic ovary syndrome. Current evidence suggests that using metformin in these settings does not raise short-term safety concerns. Some studies show that metformin reduces maternal weight gain and lowers the incidence of large-for-gestational-age (LGA) infants. Despite these benefits, the broader adoption of metformin is limited by concerns about its ability to cross the placenta, resulting in foetal concentrations comparable to maternal levels. *In utero* exposure to metformin has been shown to induce mitochondrial and epigenetic alterations in animal and *ex vitro* studies. These changes have been linked to childhood obesity, altered adiposity markers and future cardiovascular disease (CVD) risk. Both LGA and small-for-gestational-age (SGA) neonates have an increased risk of future CVD. Metformin may offer protection by reducing the incidence of LGA births; however, an increase in SGA rates, reported in some studies, could offset this potential benefit. SGA infants who experience rapid catch-up growth are particularly vulnerable. It remains unclear whether the altered growth trajectory in offsprings of metformin-treated mothers increases future CVD risk. The final risk likely reflects a multifactorial interaction involving maternal metabolic status, degree of glycaemic control, placental function, the mitigating effect of metformin on LGA, and a predisposition to SGA and childhood adiposity. Longitudinal prospective studies are essential to understand the long-term cardiovascular implications of foetal metformin exposure. Applying precision medicine to identify women likely to benefit from metformin, offers a rational strategy to optimize pregnancy outcomes.

Gestational diabetes (GD) is a heterogeneous disorder that arises when pancreatic insulin secretion fails to compensate for the insulin resistance of pregnancy. A disbalance in either component, insufficient secretion or excessive resistance, can lead to GD and reflects its variable pathophysiological spectrum.^1^ Additionally, hyperglycaemia in pregnancy could be because of pre-existing type 1 diabetes or type 2 diabetes (T2D). Besides, overt diabetes can be identified during first-trimester screening. Overt diabetes is diagnosed during pregnancy when a woman meets non-pregnant diagnostic criteria for diabetes.^2,3^

The prevalence of both GD and pre-existing diabetes in pregnancy has increased in recent years, mainly due to the global obesity epidemic.^4,5^ Increasing maternal age, sedentary lifestyles and dietary shifts towards high-calorie, processed foods have further contributed to this trend.^6^ According to the International Diabetes Federation estimates for 2024, 19.7% of pregnancies were affected by some form of hyperglycaemia, impacting around 23 million live births globally. GD (79.2%) accounted for the majority of these, followed by pre-existing diabetes (11%) and overt diabetes identified first during pregnancy (9.9%).^7^

Furthermore, early gestational diabetes (eGD), or early abnormal glucose metabolism (EAGM), has been recognized as another spectrum of hyperglycaemia in pregnancy.^2,8^ Hyperglycaemia that is diagnosed in early pregnancy, typically before 24 weeks of gestation, but does not meet the criteria for overt diabetes has been labelled as eGD.^8,9^ The prevalence of eGD varies from 9.2% to 43.4%, depending on the criteria and the ethnicity.^10,11^ The conventional forms of hyperglycaemia in pregnancy, as well as eGD, negatively impact maternal and foetal outcomes.^12–14^ Lifestyle changes and insulin are recommended to improve outcomes in GD and overt diabetes.^12,15^ Evolving evidence suggests that treating eGD might also be beneficial.^16,17^ Insulin has traditionally been the pharmacotherapy of choice for hyperglycaemia during pregnancy; however, the use of metformin has increased in recent years. Metformin has gained popularity as an alternative or adjunct to insulin due to its ease of administration, affordability, minimal risk of hypoglycaemia and reasonable safety profile.^18^ Other than hyperglycaemia, metformin might have a beneficial role in women with polycystic ovary syndrome (PCOS).^19^

While metformin is a convenient option, there are concerns regarding transplacental passage and its long-term safety.^20,21^ Studies indicate the possibility of an increase in the risk of spontaneous preterm birth and small-for-gestational-age (SGA) infants.^22,23^ There is ongoing debate about the connection between metformin and childhood adiposity.^24,25^ Furthermore, there are questions about whether SGA infants or children with higher adiposity, following foetal metformin exposure, might face an increased risk of metabolic and cardiovascular disease (CVD) in adulthood.^26–28^ While some guidelines recommend metformin as a first-line treatment, others advise caution regarding its use during pregnancy due to the lack of long-term safety data.^15,29^

Though metformin results in favourable short-term safety, several unresolved aspects regarding long-term effects and optimal clinical usage exist. There are uncertainties about the timing of exposure, duration of treatment and optimal dose in pregnancy. Foetal exposure to metformin could induce epigenetic interactions that could be both protective and potentially concerning and is an area of ongoing research.^30,31^ In this narrative review, we briefly discuss the potential uses of metformin during pregnancy, examine its possible connection to childhood obesity and explore whether this link could lead to an increased risk of CVD in adulthood.

## Literature search strategy

For this narrative review, we searched PubMed, Scopus, Cochrane Library and Google Scholar for articles published in English before April 2025. The search terms were a combination of ‘type 2 diabetes mellitus’, ‘gestational diabetes mellitus’, ‘overt diabetes in pregnancy’, ‘early gestational diabetes mellitus’, ‘polycystic ovary syndrome’, ‘childhood adiposity’, ‘small-for-gestational-age’, ‘large-for-gestational-age’, ‘cardiovascular disease’ and ‘metabolic syndrome’ with ‘metformin’. The abstracts were screened, and the full text of all relevant articles was studied. The references of the selected papers were also examined.

## Transplacental passage of metformin

Metformin freely crosses the placenta, with foetal serum levels similar to or higher than maternal levels.^20,21,32,33^ The average foetal:maternal ratio for metformin is 1.5:1.^34^ The specific transporter responsible for placental passage in humans remains unknown. *Ex vivo* studies suggest that a carrier that transfers cationic compounds bidirectionally is responsible, with a greater transfer rate from the foetus to the maternal circulation.^35^ *Ex vivo* models and experimental studies suggest that the foetal metformin uptake may be mediated by organic cation transporter (OCT) type 3 (OCT3), expressed on the syncytiotrophoblast basal membrane and foetal capillaries.^36^ Rodent studies suggest that OCT3 and multidrug and toxin extrusion 1 protein mediate the transfer.^37^ Human placental *ex vivo* studies demonstrate that the inhibition of OCT does not alter transplacental metformin passage, suggesting the possible role of other pathways.^38^ Metformin also did not affect placental glucose uptake and transport in the human single-cotyledon model.^39^

## Metformin use in pregnancy and foeto-maternal outcomes

Metformin, a cornerstone of treatment for T2D, primarily lowers blood glucose by reducing hepatic glucose production, though the precise molecular mechanism remains a topic of debate. It has traditionally been presumed to activate adenosine monophosphate-activated protein kinase (AMPK) via inhibition of mitochondrial complex I, although this effect is typically observed at supratherapeutic concentrations.^40^ More recent evidence suggests that at clinically relevant doses, metformin may act through a redox-dependent pathway.^41^ In addition to its hepatic effects, metformin might improve peripheral glucose uptake, modulate gut microbiota and gastrointestinal function and favourably influence lipid metabolism.^42^

Metformin has been used in GD, pregnancies with T2D, eGD and PCOS. Across the whole spectrum, metformin use did not result in any major short-term safety concerns and, in many trials, showed benefits by optimizing maternal weight and glycaemic control and reducing large-for-gestational-age (LGA) rates, caesarean delivery and neonatal hypoglycaemia.

### Gestational diabetes

The efficacy and short-term safety of metformin in GD have been demonstrated in several randomized controlled trials (RCTs).^43–47^ The landmark Metformin in Gestational Diabetes (MiG) trial (Metformin in gestational diabetes: follow up of offspring of mothers treated with insulin compared with metformin; Australian New Zealand Clinical Trials Registry number: ACTRN12605000311651) compared metformin with supplemental insulin versus insulin alone.^43^ The primary outcome, a composite of neonatal hypoglycaemia, need for phototherapy, respiratory distress, birth trauma, 5 min Apgar score <7 or prematurity, did not differ in these two arms. Maternal weight gain after enrolment, a secondary outcome, was lower in the metformin group (0.4 ± 2.9 kg versus 2.0 ± 3.3 kg; p<0.001). Birth weight, neonatal anthropometrics and rates of LGA infants were similar. The rates of severe neonatal hypoglycaemia [<28.8 mg/dL (1.6 mmol/L)] were lower in the metformin group. Other RCTs have shown a reduction in the rates of preeclampsia, pregnancy-related hypertension and admissions to the neonatal intensive care unit (NICU). However, some studies observed a lower mean birth weight and higher rates of SGA.^44–48^ Treatment failure with metformin monotherapy occurred in 14–46% of cases.^46,49–51^

A meta-analysis of 24 RCTs suggested that metformin reduced the risks of preeclampsia, induction of labour, caesarean delivery, neonatal hypoglycaemia, NICU admission, macrosomia and LGA infants. Conversely, metformin did not increase the risk of SGA.^52^ An earlier meta-analysis found lower birth weight in the metformin group than in the insulin group.^53^ Thus, current evidence indicates that metformin can decrease the chance of LGA and reduce maternal weight gain, but it can also increase the probability of SGA.

The DECIDE trial (DECIDE: A Comparative Effectiveness Trial of Oral Metformin Versus Injectable Insulin for the Treatment of Gestational Diabetes; ClinicalTrials.gov identifier: NCT06445946), a large, pragmatic, open-label RCT comparing the effectiveness, safety and patient experience of oral metformin versus insulin in managing GD, is currently underway.^54^ It also investigates patient preferences and the impact on offspring health up to 2 years after birth. The study plans to enrol over 1,572 participants across multiple centres in the USA.

### Type 2 diabetes

The Metformin in women with type 2 diabetes in pregnancy (MiTy) trial (Metformin in Women With Type 2 Diabetes in Pregnancy Trial; ClinicalTrials.gov identifier: NCT01353391) investigated the effects of adding metformin to a standard insulin regimen.^22^ The study concluded that metformin-exposed infants had a lower mean birth weight, were less likely to have a birth weight >97th percentile and had reduced adiposity measures. In the metformin group, 13% of infants were SGA, compared with 7% in the placebo group. Moreover, metformin-treated women showed better glycaemic control, with a lower mean glucose level and glycated haemoglobin A1c (HbA1c) at 34 weeks and required less insulin. They also gained less weight and had fewer caesarean births.

Another RCT with 794 participants, investigating the effects of metformin in individuals with pre-existing T2D or diabetes diagnosed before 23 weeks of pregnancy, reported lower rates of LGA infants among those treated with the drug; however, all other outcomes were similar between the two groups.^44^ A smaller study (n=206) found less maternal weight gain and pregnancy-induced hypertension but higher rates of SGA with metformin. Neonatal hypoglycaemia and NICU stay were less in the metformin group.^55^

### Early gestational diabetes

EAGM or eGD is another spectrum of hyperglycaemia in early pregnancy. In eGD, the glycaemic thresholds before 20 weeks meet the criteria to define GD at 24–28 weeks but do not reach the level for overt diabetes.^2,8^ Evolving evidence favours initiating treatment for eGD, especially at higher thresholds.^16,17^ The findings are primarily driven by the Treatment of Booking Gestational Diabetes (TOBOGM) trial (The Treatment Of BOoking Gestational diabetes Mellitus Study: Evaluating the impact on obstetric outcomes of immediate versus delayed care for gestational diabetes diagnosed at booking, Australian New Zealand Clinical Trials Registry number: ACTRN12616000924459), the only multicentre RCT on eGD published to date.^16^ Metformin was used in 23.6% of women in the immediate treatment group and 10.4% in the delayed treatment group, although the effects of individual drugs have not yet been reported. Further studies are necessary to assess the impact of metformin in eGD.

### Polycystic ovary syndrome

Metformin has been hypothesized to reduce pregnancy complications in PCOS. A multicentre, double-blind RCT, PregMet2 (A Prospective, Randomized, Double-blind, Multi-centre Study, Where the Possible Effect of Metformin to Prevent Late Miscarriage and Preterm Delivery is Studied in Women With Polycystic Ovary Syndrome (PCOS), ClinicalTrials.gov identifier: NCT01587378), had demonstrated that metformin treatment from the late first trimester until delivery might reduce the risk of late miscarriage and preterm birth, but it does not prevent GD. There was reduced maternal weight gain from inclusion until 36 weeks of gestation in the metformin group.^19^ However, no difference in hypertensive disorders, the occurrence of GD, birth weight or adverse neonatal or maternal outcomes was noted. Some of the other RCTs also failed to show any benefit.^56,57^

A meta-analysis of 17 studies (both RCT and non-RCT) with 2,899 participants demonstrated reduced incidence of early miscarriage, preterm delivery, preeclampsia, GD, the need for insulin treatment and maternal weight gain.^58^ Another meta-analysis of 11 RCTs found that metformin does not lower the risk of GD in high-risk individuals with obesity, PCOS or pre-existing insulin resistance.^59^ Another meta-analysis of six RCTs involving 1,229 participants showed benefit only in the risk of preterm delivery but did not affect the incidence of GD, miscarriage, preeclampsia and birthweight.^60^

### Current recommendations on metformin use in pregnancy

While there is heterogeneity in the outcomes across the spectrum of hyperglycaemia with metformin, the reasons for this discrepancy remain unclear. Factors such as the timing of metformin initiation, dosage, additional therapies, the level of glycaemic control, diagnostic criteria and the disease process itself may be responsible. Currently, there is no consensus about the use and safety of metformin in pregnancy.

The American Diabetes Association (ADA) recommends insulin as the primary pharmacotherapy for GD and cautions about metformin as an alternative, as it crosses the placenta and has uncertain long-term effects.^15^ The National Institute for Health and Care Excellence advocates metformin for GD if lifestyle modification fails.^29^ The American College of Obstetricians and Gynaecologists suggests that metformin is a reasonable alternative in women who deny or are unable to safely administer or afford insulin.^61^ For PCOS, the ADA advises discontinuing metformin by the end of the first trimester if used for conception.^15^ Although short-term studies indicate metformin is generally safe, concerns exist about its association with lower birth weight and increased childhood adiposity. Given the limited long-term safety data, guidelines emphasize careful consideration of benefits and risks, with individualized treatment approaches to optimize maternal and foetal outcomes. Current recommendations about metformin use in pregnancy are summarized in *Table 1*.^15,16,19,22,24,29,43,44,53,55,56,61–65^

## Foetal metformin exposure and small-for-gestational-age: Clinical outcome

Metformin’s use in pregnancy presents a complex balance between maternal glycaemic benefits and potential foetal growth restriction.^66^ Metformin decreases the likelihood of LGA, macrosomia and caesarean section but may be associated with an increased risk of SGA.^22,52,53^ This is concerning as infants born SGA are at risk of CVD in adulthood, influenced by intrauterine growth patterns, postnatal growth trajectories and epigenetic factors.^67,68^ Studies involving women with pre-existing T2D showed a stronger link to SGA infants. The tendency towards smaller neonates was not consistently seen in GD and PCOS.^19,22,43^

### Type 2 diabetes

The MiTy trial studied the effect of adding metformin (2,000 mg/day between 6 and 22 weeks of gestation) to a standard insulin regimen in women with T2D. A higher incidence of SGA infants in the metformin group (13%) compared with the placebo group (7%) [relative risk (RR) 1.96; p=0.026] was observed.^22^ A sub-analysis suggested that metformin use in the presence of comorbidities such as chronic hypertension and nephropathy increased the risk of SGA.^66^ Due to the concerns about growth restriction in cases of placental insufficiency, the ADA recommends that metformin should be avoided in pregnancies with hypertension, preeclampsia or in those at risk of intrauterine growth restriction (IUGR), such as multiple gestations and placental abnormalities.^15^

In contrast, in the Medical Optimization of Management of Overt Type 2 Diabetes in Pregnancy (MOMPOD) (Medical Optimization of Management of Type 2 Diabetes Complicating Pregnancy; ClinicalTrials.gov identifier: NCT02932475), the SGA rates did not differ in metformin and placebo groups.^44^ The trial included women with pre-existing T2D or diabetes diagnosed early in pregnancy who were randomized to receive either metformin 1,000 mg twice a day or placebo from enrolment (11–23 weeks) through delivery.

**Table 1:** Summary of maternal and neonatal outcomes of metformin use in pregnancy and society recommendations^15,16,19,22,24,29,43,44,53,55,56,61–65^

	Maternal outcomes	Neonatal outcomes	Society recommendations
GD	Reduced maternal weight gain during pregnancy, reduction in dose of supplemental insulin, fewer caesarean deliveries, lower incidence of pregnancy-induced hypertension^43,53^	Reduced neonatal hypoglycaemia, lower incidence of macrosomia or LGA, increased preterm births (clinically insignificant)^24,43,53,62^	ADA: insulin is preferred as the first-line agent for the management of GD; avoid metformin in pregnant women with hypertension, preeclampsia or those at risk for intrauterine growth restriction^15^
			CDA: metformin can be considered as an alternative therapy^63^
			NICE: metformin is considered a first-line therapy, while insulin is reserved for special circumstances^29^
			ACOG: insulin is the preferred first-line therapy; metformin is a reasonable alternative in women who deny or are unable to safely administer or afford insulin therapy^61^
Overt diabetes (pre-existing/undiagnosed)	Reduced maternal weight gain, reduction of insulin dose requirement, fewer caesarean births, no difference in hypertensive disorder^22,55^	Decrease in birth weight, reduction in macrosomia rates, increased incidence of SGA decreased neonatal hypoglycaemia lower odds for LGA^22,44,55^	ADA: insulin is preferred over metformin as the first-line agent^15^
			CDA: insulin is preferred over metformin therapy^63^
			Italian: metformin is considered a second-line agent or adjunct to insulin, especially if BMI >35 kg/m^2^, in PCOS, in women undergoing ART^64^
			NICE: metformin can be continued as the first-line therapy^29^
			ACOG: insulin is the preferred first-line therapy; metformin is a reasonable alternative in women who deny or are unable to safely administer or afford insulin^65^
eGD	No difference in maternal weight gain, caesarean delivery rates or hypertensive disorders in the immediate treatment group; subgroup analysis of metformin on maternal outcomes not available^16^	A modest reduction of composite neonatal outcomes, primarily led by lower rates of neonatal respiratory distress in the immediate treatment group; no difference in birth weight and neonatal lean body mass; no data to demonstrate metformin’s effect on outcomes^16^	No recommendations at present
PCOS	Reduced maternal weight gain; no difference in hypertensive disorders in pregnancy or GD, or additional need for insulin therapy^19,56^	Reduced composite primary outcome of late miscarriage and preterm delivery in the metformin group; no difference in birth weight, APGAR score, neonatal death^19^	ADA: metformin, when used for ovulation induction in PCOS, should be discontinued by the end of the first trimester^15^

*ACOG = American College of Obstetricians and Gynecologists; ADA = American Diabetes Association; ART = assisted reproductive technology; BMI = body mass index; CDA = Canadian Diabetes Association; eGD = early gestational diabetes; GD = gestational diabetes; IUGR = intrauterine growth restriction; LGA = large-for-gestational-age; NICE = National Institute for Health and Excellence; PCOS = polycystic ovary syndrome; SGA = small-for-gestational-age.*

### Gestational diabetes

A recently published meta-analysis of 24 RCTs with 4,934 pregnancies did not show an association between metformin and SGA (RR 0.93; 95% CI: 0.71, 1.22; p=0.62).^52^ In a secondary analysis of the EMERGE trial (A Randomised Placebo Controlled Trial of the Effectiveness of Early MEtformin in Addition to Usual Care in the Reduction of Gestational Diabetes Mellitus Effects [EMERGE]; ClinicalTrials.gov identifier: NCT02980276) where early metformin (500–2,500 mg/day) was initiated by 28 weeks in addition to the usual care, the likelihood of SGA in the metformin and placebo groups (5.7% versus 2.7%) was comparable.^69^ Hypertensive disorders of pregnancy, particularly preeclampsia, were strongly associated with SGA infants, independent of metformin exposure. Metformin-exposed SGA infants did not exhibit a more severe SGA phenotype than placebo-treated infants.^69^ Overall, SGA rates are similar in women with GD receiving metformin or placebo, though a tendency towards low birth weight (LBW) has been reported in cohorts exposed to metformin.

### Early gestational diabetes

In the TOBOGM trial, birth weight was slightly lower in the immediate-treatment group compared with the control group (3,258 g versus 3,343 g; adjusted difference: -72.1 g; 95% CI: -127.6, -16.6g). However, the difference in SGA rates between the treatment groups (12.0% in the immediate-treatment group versus 9.2% in the control group) did not reach statistical significance. While metformin was used more frequently in the early-treatment group (23.6% versus 10.4%), its specific impact on birth weight was not separately analysed. Exploratory sub-analysis suggests that early treatment in women at a lower glycaemic threshold increased the risk of SGA infants. Still, again, information to connect the findings with metformin was unavailable.^16^

**Figure 1:**
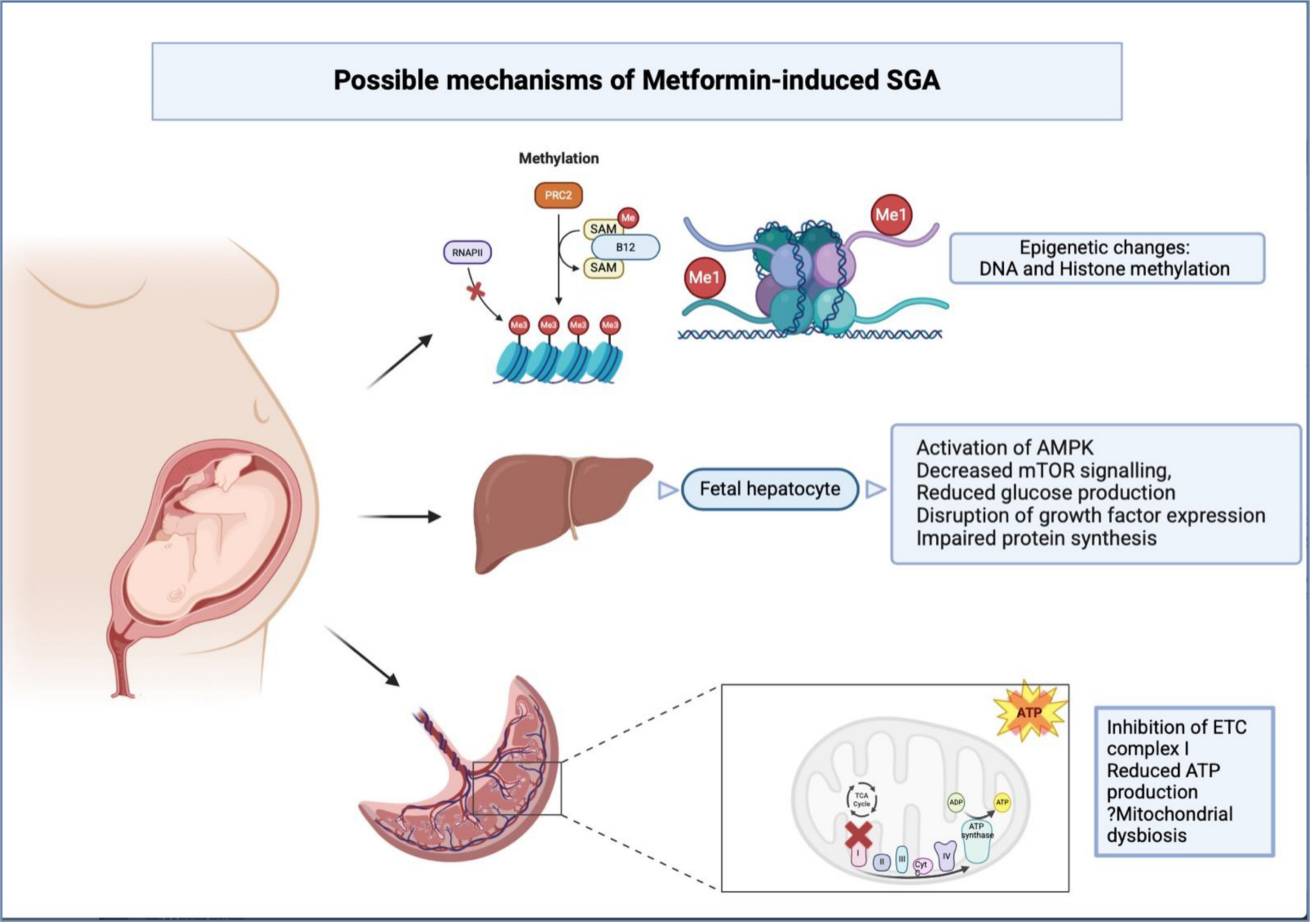
Possible mechanisms of small-for-gestational-age in foetus exposed to metformin

*AMPK = adenosine monophosphate-activated protein kinase; DNA = deoxyribonucleic acid; ETC = electron transport chain; ME = methylation; ME1 = mono-methylation; ME3 = trimethylation; B12 = vitamin B12; mTOR = mechanistic target of rapamycin; PRC2 = polycomb repressive complex 2; RNAP II = RNA polymerase II; SAM = S-adenosyl methionine; SGA = small-for-gestational-age.*

### Polycystic ovary syndrome

Metformin treatment during pregnancy did not alter the birth weight of infants born to mothers with PCOS.^19,56^ A meta-analysis, where only two studies reported IUGR, demonstrated that metformin, in fact, had a protective effect on SGA.^70^

## Possible mechanisms of metformin-induced small-for-gestational-age

The exact pathophysiological mechanism of metformin-induced SGA remains unclear. Potential factors include disturbances in mitochondrial function and nutrient sensing, maternal metabolic shifts, foetal energy limitations and epigenetic modifications, as illustrated in *Figure 1*.

### Placental metabolism

Placental energy production is crucial in foetal growth and metabolic programming, as it facilitates oxygen delivery, nutrient transport and hormonal signalling. The placenta relies on maternal glucose to sustain its high metabolic activity and support foetal development. Since metformin is known to inhibit complex I of the electron transport chain, it may lead to reduced oxygen consumption, adenosine triphosphate (ATP) production and mitochondrial-dependent energy metabolism in the trophoblast cells, raising concerns for placental insufficiency.^71^ *Ex vivo* and *in vitro* studies suggest that metformin reduces basal mitochondrial respiration in trophoblasts in a dose-dependent manner. Metformin-treated trophoblasts show decreased ATP production but maintain integrity, as indicated by reduced proton leak. Additionally, a reduction in oxidative stress at the gene expression level due to lower mitochondrial respiration was observed.^34^ Conversely, a recent study suggested that metformin alleviated placental inflammation by promoting mitochondrial biogenesis and reducing pyroptosis through the reactive oxygen species/thioredoxin-interacting protein/nucleotide-binding oligomerization domain-like receptor protein three pathway. Pyroptosis is a programmed cell death pathway marked by the release of strongly proinflammatory cytokines. The effect of metformin on mitochondrial function and oxidative stress appears to be influenced by the underlying maternal condition, with beneficial effects in conditions such as obesity, GD and gestational hypertension but may have adverse effects in healthy, lean women.^72^ However, the clinical relevance of these findings needs to be explored further.

### Foetal energy restriction and impaired protein synthesis

Apart from impaired placental bioenergetics, animal studies have demonstrated probable effects of metformin on foetal hepatocytes, leading to foetal energy restriction. The foetal liver expresses organic cation transporter type 1, enabling metformin uptake *in vivo*. Metformin activates AMPK, decreases the mammalian target of rapamycin signalling, reduces glucose production and lowers oxygen consumption. Moreover, it triggers metabolic stress pathways and disrupts growth factor expression. These catabolic pathways impair protein synthesis and reduce foetal growth in metformin-exposed pregnancies.^73^

### Foetal pancreatic β-cell development and insulin secretion

An *in vitro* study found that differentiation of human embryonic stem cells to pancreatic β-cells can be inhibited by metformin. Metformin exposure during pancreatic β-cell differentiation reduced the expression of key pancreatic genes affecting insulin secretion. The study additionally demonstrated that metformin downregulated genes related to hormone regulation, transport and secretion. Despite upregulating some mitochondrial genes, metformin reduced mitochondrial respiration and glycolysis, leading to lower ATP generation, which is crucial for β-cell function. Functional tests showed that metformin inhibited insulin secretion.^74^

In INS1 (832/13) β-cells, a clonal subline of the rat insulinoma cell line INS-1 developed to serve as a model for pancreatic β-cell function, metformin induced metabolic deceleration by reducing glucose oxidation, oxygen consumption and ATP production. However, despite this slowdown, insulin secretion remained partially preserved at high glucose levels.^75^ Foetal insulin levels were, however, not affected by metformin exposure in PCOS pregnancies.^76^ Whether reduced foetal growth and lower neonatal hypoglycaemia, alongside higher rates of SGA infants, reflect metformin’s impact on foetal insulin secretion remains a hypothesis that warrants further exploration.

### Epigenetic changes

Exposure to metformin during foetal development may lead to changes in metabolic programming, potentially through epigenetic modifications that influence gene expression in critical organs such as the pancreas, liver, central nervous system and heart.^31^ These changes may influence organogenesis, metabolic function and long-term health outcomes in the offspring. Studies have suggested that metformin disrupts one-carbon metabolism, impairs histone acetylation via AMPK–sirtuin activation, affects DNA and histone methylation through vitamin B12 deficiency and modulates nitric oxide synthase, potentially altering developmental programming through epigenetic mechanisms.^77^

### Vitamin B12 deficiency and folate disbalance

Metformin use during pregnancy is associated with vitamin B12 and folate deficiencies, probably due to intestinal malabsorption secondary to bacterial overgrowth and intrinsic factor deficiency.^78^ Maternal deficiencies pose risks for the foetus, including neural tube defects, cognitive impairments and irreversible neurological damage in infants.^79^ The MiG trial found that metformin-treated women with GD experienced greater vitamin B12 depletion than those on insulin, with the degree of depletion being linked to the duration of therapy. Nevertheless, biomarkers of active vitamin B12 and homocysteine remained similar between the groups, raising doubts about the role of vitamin B12 depletion in causing foetal growth restriction.^80^

Additionally, folate over-supplementation can further compound the effect of vitamin B12 deficiency by altering one-carbon metabolism and raising homocysteine levels. Elevated homocysteine levels have been linked to greater oxidative stress, increased apoptosis in trophoblast cells and impaired pancreatic β-cell activity. The connection between excessive folic acid intake, disrupted one-carbon metabolism and the development of GD, though, remains incompletely defined.^81^

### Gender divergence

In an obese murine model of pregnancy exposed to metformin, male offspring exhibited increased adiposity and adipose tissue inflammation, whereas female offspring remained unaffected, indicating sex-specific metabolic programming effects.^82^ In another murine model, metformin exposure led to lower birth weight but more weight gain from 9 weeks of birth, resulting in more mesenteric fat under a high-fat diet. Male offspring showed impaired glucose tolerance, elevated fasting glucose and reduced glucose transporter type 4 mRNA in epididymal fat.^83^ The mechanisms driving these sex differences are not fully understood, but oestrogen’s anti-inflammatory effects may play a role in the relative protection observed in females.^84^

## Childhood metabolic and adiposity profile in pregnancies with gestational diabetes and metformin exposure

The heterogeneity in childhood obesity and metabolic parameters following treatment with metformin for GD has been a subject of debate. The Metformin in Gestational Diabetes: The Offspring Follow-Up (MiG TOFU) study (Australian New Zealand Clinical Trials Registry number: ACTRN12605000311651) followed up children from Auckland and Adelaide cohorts at 2 and at 7–9 years after *in utero* exposure to metformin for GD treatment.^85,86^ At the 2-year follow-up, children exposed to metformin showed higher subcutaneous fat measurements, such as increased mid-upper arm circumferences and skinfold thickness, than those born to mothers treated with insulin. However, the two groups had similar total fat mass and body fat percentage.

In the Adelaide cohort, there were no differences in body composition or metabolic markers (fasting plasma glucose, glycosylated haemoglobin, triglyceride, cholesterol, insulin, liver enzymes, leptin, adiponectin and biochemical markers of insulin resistance) between the two groups at 7 years. In contrast, in the Auckland cohort, metformin-exposed children were larger in weight, body mass index (BMI), arm and waist circumference, waist-to-height ratio, triceps skinfold thickness and abdominal fat volume by magnetic resonance imaging (all p=0.05), but had similar total and abdominal fat percentages at 9 years. The infants in the Adelaide group, exposed to higher nutrient load (as measured by high maternal glucose levels) *in utero*, could be protected by metformin, as they did not develop increased obesity or glucose intolerance as they grew. This aligns with metformin’s known mechanisms, which help mitigate the effects of excess nutrient supply, improving metabolic outcomes in offspring. On the other hand, the Auckland cohort had a heterogeneous population and less well-matched treatment groups compared with the Adelaide cohort, and women randomized to metformin had a higher BMI than those in the insulin group.^86^ These factors could account for the disparity in results between the two groups.

In a meta-analysis by Tarry-Adkins et al., metformin-exposed neonates had lower birth weight (by 108 g) and a lower risk of macrosomia and LGA births.^24^ However, by 18–24 months, metformin-exposed infants were 0.44 kg heavier, and by 5–9 years, they had a higher BMI. In the Born in Bradford study, the offspring of mothers with GD treated with metformin and insulin had similar growth trajectories from birth to 60 months.^87^ The metformin group had a lower birthweight z-score than those without GD but showed faster height growth from 17 to 60 months, reaching a comparable BMI z-score by 60 months.

On the contrary, in a Finnish cohort, there were no differences in anthropometric measures, body composition, adiposity, liver fat or inflammation markers between children of insulin- and metformin-treated mothers at 9 years. Boys exposed to metformin had higher high-density lipoprotein (HDL) cholesterol levels, higher adiponectin levels and a lower leptin/adiponectin ratio than those in the insulin group, suggesting a possible metabolic benefit.^88,89^ The summary of studies describing outcomes in children following maternal treatment with metformin for GD is tabulated in *Table 2*.^26,85–90^ The findings are discordant and call for more studies to understand the impact of factors that influence the development of childhood adiposity.

**Table 2:** Summary of major trials analysing the effect of foetal metformin exposure in pregnancy^26,85–90^

Study (year of publication)	Study type	Parameters studied	Outcome
MiG TOFU (2011)^85^	Longitudinal follow-up study of the offspring of women with GD recruited into a prospective RCT comparing metformin with insulin	Anthropometry, BIA, DXA	Larger MUAC (17.2 ± 1.5 versus 16.7 ± 1.5 cm; p=0.002) and subscapular (6.3 ± 1.9 versus 6.0 ± 1.7 mm; p=0.02), biceps skinfolds (6.03 ± 1.9 versus 5.6 ± 1.7 mm; p=0.04) in the metformin group; total fat mass and percentage body fat assessed by BIA (n=221) and DXA (n=114) were not different
MiG TOFU (2018)^86^	Longitudinal follow-up study of the offspring of women with GD recruited into a prospective randomized trial comparing metformin with insulin	Anthropometry, BIA, DXA, MRI, fasting plasma glucose, glycosylated haemoglobin, liver enzymes, leptin, adiponectin, triglyceride, cholesterol and insulin in children at 7 years (Adelaide cohort) and children at 9 years (Auckland cohort)	Adelaide cohort: no differences in body composition or metabolic markers; Auckland cohort: metformin-exposed children were larger in weight, BMI, arm and waist circumference, waist-to-height ratio, triceps skinfold thickness and abdominal fat volume by MRI (all p=0.05) but had similar total and abdominal fat percentages at 9 years
Paavilainen et al. (2022)^88^	Two-centre open label RCT	Anthropometrics, blood pressure, lipoproteins and oral glucose tolerance tests in children at 9 years of age	Offspring in the metformin group had higher HDL-cholesterol (1.72 versus 1.54 mmol/L; p=0.039) but lower LDL-cholesterol (2.39 versus 2.58 mmol/L; p=0.046) and apolipoprotein B (0.63 versus 0.67 g/L; p=0.043) than the offspring in the insulin group; the difference in HDL cholesterol was significant only in boys (p=0.003); The 2-h glucose value in the 75 gm glucose 2-h OGTT was 10.8 mg/dL (0.6 mmol/L) lower in boys from the metformin group than the insulin group (p=0.015)
Paavilainen et al. (2023)^89^	Two-centre open label RCT	Anthropometrics, markers of the low-grade inflammation, adipocytokines, abdominal MRI, MRS and whole body DXA	Adiponectin concentration was higher in children in the metformin group than the insulin group in boys only (median 12.13 versus 7.50 μg/ml, p<0.001). Leptin:adiponectin ratio was lower in boys in the metformin group than in the insulin group (median 0.30 versus 0.75; p=0.016)
BiB (2023)^87^	Longitudinal prospective birth cohort study	Offspring growth outcomes: weight-for-age, height-for-age and BMI-for-age z-scores from 0 to 60 months, according to the WHO Child Growth Standards	OGD–metformin and OGD–insulin had similar growth trajectories from birth to 60 months; OGD–metformin had a lower birthweight z-score than those without GD, but showed faster height growth from 17 to 60 months, reaching a comparable BMI z-score to No-GD by 60 months
MiTy Kids^90^	Longitudinal follow-up study of children born to mothers with type 2 diabetes who participated in the MiTy trial	Anthropometrics, glucose, insulin, liver function, lipids, adiponectin and leptin at 24 months	At 24 months, there was no significant difference in BMI z-score between the metformin and placebo groups (mean difference = 0.07; 95% CI: -0.31, 0.45; p=0.72) or in the sum of skinfolds (mean difference = 0.8 mm; 95% CI: -0.7, 2.3; p=0.31); in males, BMI trajectory differed significantly by treatment (p=0.048), with higher BMI in the metformin group from 6 to 24 months
PedMet^26^	Longitudinal follow-up study of children aged 5–10 years whose mothers had participated in the PregMet trial	Anthropometrics,: adiponectin, cholesterol, triglycerides, HDL-cholesterol, non-HDL cholesterol, alanine transaminase, glucose, HbA1c, insulin, c-peptide and HOMA-IR	Children exposed to intrauterine metformin had significantly higher BMI z-scores (difference = 0.41; 95% CI: 0.03, 0.78; p=0.03), greater waist-to-height ratio z-scores (difference = 0.36; p=0.02) and higher obesity prevalence (17% versus 1%; p=0.001) at 5–10 years compared with placebo. No significant differences were observed in metabolic markers, blood pressure or heart rate

*BIA = bioimpedance analysis; BiB = Born in Bradford; BMI = body mass index; DXA = dual-energy X-ray absorptiometry; GD = gestational diabetes; HbA1c = glycated haemoglobin; HDL = high-density lipoprotein; HOMA-IR = homeostatic model assessment for insulin resistance; MiG TOFU = Metformin in Gestational Diabetes: The Offspring Follow-Up; MiTy = metformin in women with type 2 diabetes in pregnancy; MRI = magnetic resonance imaging; MRS = magnetic liver spectrometry; MUAC = mid-upper arm circumference; No-GD = offspring of mothers without; OGD-insulin = offspring of mothers with GD treated with insulin; OGD-metformin = offspring of mothers with GD treated with metformin; OGTT = oral glucose tolerance test; PedMet = Intrauterine metformin exposure and offspring cardiometabolic risk factors; PregMet = Metformin to Prevent Late Miscarriage and Preterm Delivery in Women With Polycystic Ovary; RCT = randomized controlled trial; WHO = World Health Organization.*

## Childhood metabolic and adiposity profile in pregnancies with type 2 diabetes and metformin exposure

The MiTy trial demonstrated no differences in the primary neonatal outcomes, with the addition of metformin actually being beneficial in several key aspects. However, infants in the metformin group had lower birth weight and less adiposity but were more likely to be SGA (13% versus 7%, p=-0.026).^22^ The offspring follow-up trial, MiTy Kids (ClinicalTrials.gov identifier: NCT01832181), demonstrated that at 24 months, there was no difference between the metformin and placebo groups in BMI z-score or skinfold thickness, indicating no impact on the body composition.^90^ While BMI trajectories were similar overall, a sex-specific effect was observed. Males in the metformin group had a higher BMI between 6 and 24 months (p=0.048). Further studies are needed to confirm these findings and assess if there is a gender-related divergence in the outcome.

## Childhood metabolic and adiposity profile in polycystic ovary syndrome pregnancies exposed to metformin

The Intrauterine metformin exposure and offspring cardiometabolic risk factors (PedMet) study (ClinicalTrials.gov identifier: NCT00159536) examined the long-term effects of intrauterine metformin exposure on children’s cardiometabolic health.^26^ They followed up children aged 5–10 years whose mothers had participated in the PregMet trial.^19^ Higher BMI, higher waist-to-height ratio and waist circumference were noted in metformin-exposed children with no significant differences in metabolic markers (adiponectin, cholesterol, triglycerides, HDL-cholesterol, non-HDL cholesterol, alanine transaminase, glucose, HbA1c, insulin, c-peptide, homeostatic model assessment for insulin resistance, blood pressure or heart rate). However, a subgroup analysis according to maternal pre-pregnancy BMI showed that metformin-exposed children had higher measures of adiposity than placebo-exposed children, higher triglycerides and heart rate, and lower HDL-cholesterol only when maternal pre-pregnancy BMI was >30 kg/m^2^.^26^

(PedMet) study examined the long-term effects of intrauterine metformin exposure on children’s cardiometabolic health.^26^ They followed up children aged 5–10 years whose mothers had participated in the PregMet trial.^19^ Higher BMI, higher waist-to-height ratio and waist circumference were noted in metformin-exposed children with no significant differences in metabolic markers (adiponectin, cholesterol, triglycerides, HDL-cholesterol, non-HDL cholesterol, alanine transaminase, glucose, HbA1c, insulin, c-peptide, homeostatic model assessment for insulin resistance, blood pressure or heart rate). However, a subgroup analysis according to maternal pre-pregnancy BMI showed that metformin-exposed children had higher measures of adiposity than placebo-exposed children, higher triglycerides and heart rate, and lower HDL-cholesterol only when maternal pre-pregnancy BMI was >30 kg/m^2^.^26^

## Low birth weight and future cardiovascular risk

Barker suggested that foetal undernutrition in mid-to-late gestation leads to disproportionate IUGR, which predisposes to the development of CVD in adulthood.^91^ The link between LBW or SGA and the development of CVD, diabetes and hypertension in adulthood has been explored in several observational studies.^27,28,67,92^

### Low birth weight and cardiovascular risk

A recent meta-analysis that included over 7.6 million participants from 135 studies found that higher birth weight was associated with a lower risk of several cardiometabolic diseases later in life.^27^ In particular, each 1-kg increase in birth weight was linked to a 22% reduction in the risk of T2D, a 16.5% reduction in CVD and a 23% lower risk of hypertension. However, when examining the birth weight categories, the association with T2D and CVD was J-shaped. Infants with LBW (<2.5 kg) had a 45% increased risk of T2D and a 30% higher risk of CVD compared with those who had normal birth weight. The study also reported that each 1-kg increase in birth weight was associated with lower systolic blood pressure (about 1.36 mmHg) and lower diastolic blood pressure (about 0.33 mmHg). Similarly, another meta-analysis found a U-shaped relationship between birth weight and CVD risk. Thus, both low (<2,500 g) and high (>4,000 g) birth weight have been linked to an increased risk of CVD.^28^

### Small-for-gestational-age and cardiovascular disease

After adjusting the LBW for gestational age, it was found that SGA is an independent predictor for premature CVD and myocardial infarction. A recent binational register-based cohort investigated the link between birth weight for gestational age and gestational age with CVD risk in early adulthood.^67^ It included 3.4 million births from Sweden and Denmark, with a follow-up of 10 years. SGA, as well as preterm birth, was associated with higher CVD risk (hazard ratio [HR]: 1.38 and 1.31, respectively). Similarly, earlier studies have demonstrated a negative association between foetal growth and ischaemic heart disease.^92,93^

### Proposed mechanisms

Cardiomyocyte dysfunction, increased arterial stiffness, reduced nitric oxide availability leading to endothelial dysfunction, increased insulin-like growth factor-2-mediated cardiac hypertrophy, dysfunction of the vascular smooth cells, impaired cardiac contractility, abnormal transforming growth factor-beta signalling, reduced nephron number and epigenetic alterations are the proposed pathophysiologic mechanisms linking SGA and preterm birth to increased CVD risk.^94–98^

Term SGA and IUGR foetuses show signs of cardiac dysfunction, including impaired systolic and diastolic function, along with elevated cord blood biomarkers of cardiac stress and damage.^95^

Mouse models have suggested that IUGR may lead to a decreased anorexic response to leptin, central dysregulation of appetite and abnormal adipocyte activation, increasing their risk of developing obesity later in life.^99,100^ Similarly, animal studies have shown that altered intracellular insulin signalling pathways and reduced number of pancreatic islet cells in IUGR increase the likelihood of developing glucose intolerance.^101,102^ All these maladaptive foetal programming secondary to IUGR leads to an increased risk of developing CVD, obesity and T2D.

## Childhood adiposity and future cardiovascular risk

### Childhood adiposity and subclinical markers of vascular damage

Increased carotid intima-media thickness (cIMT) is a marker of early atherosclerosis and is measured noninvasively with ultrasound. Higher cIMT is linked to a greater cardiovascular risk and can predict future cardiovascular events. Thus, cIMT measurements are a surrogate marker for assessing early atherosclerosis.^103^ A positive correlation between childhood or adolescent BMI and truncal subcutaneous fat with subclinical markers of vascular damage, including cIMT, carotid-femoral pulse wave velocity, brachial-ankle pulse wave velocity or carotid artery stiffness, has been demonstrated.^104–111^

### Childhood adiposity and left ventricular structure

Similarly, childhood BMI and waist circumference positively correlate with increased left ventricular mass, left atrial volume and left ventricular end-diastolic volume.^112,113^ The link between childhood BMI and abnormal left ventricular geometric patterns, including concentric and eccentric hypertrophy, has been reported.^114,115^

### Childhood adiposity and cardiovascular disease

The subclinical markers translate into the development of hypertension, CVD, heart failure, stroke and increased CVD mortality in adulthood. A linear association exists between childhood adiposity and increased risk of CVD in adulthood across the entire BMI distribution, with the risk increasing with age.^116,117^ Similarly, a rise of 0.5 in BMI z-score from ages 7 to 13 in children with above-average BMI is associated with a higher risk of early ischaemic stroke in both men and women.^118^ BMI has been positively correlated with the development of early heart failure, with a 10-fold increased risk in individuals with BMI >35 kg/m^2^.^119^ These childhood alterations translated to higher morbidity and mortality, with greater childhood BMI velocity being associated with an increased rate of CVD mortality.^120^ A recent Mendelian randomization analysis showed a causal relationship between childhood obesity with CVD, heart failure, myocardial infarction and atrial fibrillation in adulthood. The contributory factors were HDL cholesterol, triglycerides, hypertension and T2D.^121^

## Can we connect the dots?

The use of metformin in pregnancy presents a complex interplay between maternal glycaemic benefits and the effects of drug exposure on the foetus. The link between foetal metformin exposure and childhood adiposity and future cardiovascular risk is an active area of research and is under scrutiny. Metformin has no major short-term safety concerns and could decrease macrosomia, LGA and caesarean section rates in GD and pre-existing T2D.^19,22,43^ The only neonatal outcome that has raised concern is a trend towards SGA babies, especially in mothers with prior diabetes and associated hypertension and nephropathy.^66^ Birthweight has a U-shaped association with cardiovascular risk, with both LGA and SGA infants predisposed to future CVD.^67^ It is still unclear whether the benefits of protection from LGA could be counter-balanced by the increased risk of SGA. However, any suggestion of IUGR should serve as a caution for metformin use in pregnancy, as the risk for SGA might get aggravated.^15^

Another area of controversy is the tendency towards childhood adiposity after foetal exposure to metformin. The results are conflicting, and there is even variation between the two cohorts in the same trial. The Adelaide cohort of the MIG TOFU trial did not show any detrimental effects from metformin exposure at 7 years, whereas, in the Auckland cohort, metformin-exposed children were larger and had higher subcutaneous but not visceral fat.^86^ A possible explanation is that metformin could be protective if maternal glycemic control is suboptimal, but it could be harmful if the foetal nutrient load is lower in conditions like placental insufficiency. Childhood adiposity carries a linear risk of developing future cardiovascular disorders, and the potential role of metformin in its causation requires further evaluation.^116,117^ A pragmatic approach would be to define the subgroup of women with hyperglycaemia inpregnancy who would benefit from metformin while identifying those where it could be harmful.

The mechanistic studies that study the effect of metformin on placental metabolism are also contradictory. While metformin could decrease placental ATP production, it also has a beneficial impact by lowering oxidative stress and inflammation.^34,71,72^ Additionally, it is not clear whether the epigenetic changes induced by metformin are protective or harmful.^31^

Metformin use in pregnancy offers benefits like improved glycaemic control and reduced LGA risk, but raises concerns about increased SGA incidence and effects on childhood adiposity and future cardiovascular risk. Conflicting evidence from studies suggests that metformin’s impact may depend on the maternal glycaemic status and foetal nutrient load. Longitudinal cohorts of infants with SGA and children with adiposity have shown a clear link to future CVD risk in studies unrelated to metformin use. It remains uncertain whether children who are SGA or who develop childhood adiposity following metformin exposure face comparable risks. Given the existing uncertainties, adopting a personalized strategy that considers maternal phenotype, comorbidities and foetal growth trajectories is crucial to optimize both short- and long-term outcomes.

## Precision medicine approach to the use of metformin in pregnancy

Maternal hyperglycaemia in pregnancy, including GD, encompasses a heterogeneous spectrum of disorders characterized by varying degrees of insulin resistance and β-cell dysfunction, which influence the therapeutic response and short- and long-term outcomes.^1^ The choice of administering metformin in such a broad group may need individualization. Metformin may be more appropriate in primarily insulin-resistant GD and potentially contraindicated in predominantly insulin-deficient GD. Thus, overweight and obesity, along with features of insulin resistance, may favour metformin use, whereas caution is warranted in leaner phenotypes.^122,123^ Ethnic variations in insulin resistance and secretion could also guide treatment decisions.^124–126^ Additionally, the risks of placental insufficiency and IUGR represent potential contraindications.^15^ Access to healthcare and affordability are important considerations, especially in resource-limited settings. The long-term impact of metformin across different phenotypes remains unclear. Future research should incorporate these factors to optimize therapeutic strategies.

## Conclusion

In summary, while metformin holds promise for improving glycaemic control and reducing adverse outcomes during pregnancy, its transplacental passage and potential long-term effects on offspring mandate scrutiny. The current evidence indicates potential benefits, such as reduced risk of LGA and maternal weight gain, as well as possible concerns, including increased SGA infants and childhood adiposity. SGA, LGA and childhood adiposity have been linked to future CVD risk in studies unrelated to hyperglycaemia or metformin use. It is currently unclear whether these findings can be extrapolated to offspring with these phenotypes exposed to metformin. CVD and metabolic disorder risk could finally be an interplay between the underlying disease, degree of hyperglycaemia, placental insufficiency, protective effect on LGA and risk of SGA. The current evidence calls for individualizing metformin use in pregnancy based on maternal phenotype, comorbidities and foetal growth patterns to ensure that the benefit-to-risk ratio remains in favour of optimal maternal and child health and favourable long-term outcome.
